# Mechanical Property Analysis of a Boom–Membrane Structure Used for Aerospace Technologies

**DOI:** 10.3390/ma17133204

**Published:** 2024-07-01

**Authors:** Shuhong Xu, Xiaojiao Yu, Yue Gao, Sicong Wang, Lining Sun

**Affiliations:** 1 School of Engineering, Applied Technology Collage of Soochow University, Suzhou 215325, China; 2 School of Mechanical and Electrical Engineering, Soochow University, Suzhou 215137, China

**Keywords:** tape spring, boom–membrane, deployed, aerospace

## Abstract

Traditional deployable truss space structures previously had upper limits on their key indicators, such as the deployed area, folded ratio and total weight, and hence, the application of new extendable mechanisms with novel deployment types is desired. Foldable extendable tape spring booms made from FRP (fiber-reinforced polymer) laminate composites and their corresponding boom–membrane structures were invented in recent years to satisfy the needs of the large-scale requirements of spacecraft, especially for antennas, solar sails and solar arrays. This paper aimed to analyze the properties of the deployed states of extendable tape spring booms and their boom–membrane structures. By establishing an analytical model of the boom and the structure, the bending stiffness, critical buckling load of the boom and the fundamental frequency of the membrane structure were acquired. To provide more guidance on the boom–membrane structure design, a geometric and material parametric study was carried out. Meanwhile, an experimental study to investigate the deployed properties of the booms and membrane structures was introduced to afford some practical verification.

## 1. Introduction and Literature Review

**Figure 1:**
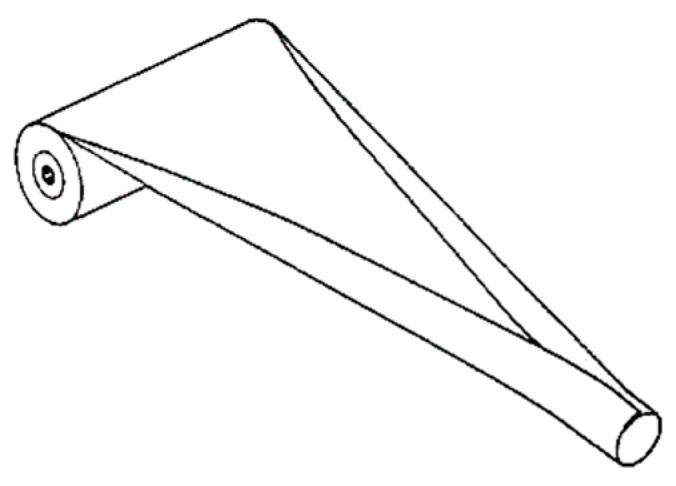
Deployable tape spring boom diagram.

Foldable deployable structure technology was previously the main method for solving the envelope limitation issues of large-size spacecraft. Recently, deployable membrane structures were introduced to replace the traditional truss morphing mechanisms in space because of their incomparable advantages such as light weight, high folded ratio and large deployed area. In terms of deployable membrane mechanisms, it was difficult to form smooth and accurate configurations with the inflatable structures after being deployed in orbit, and the ring-shaped truss–membrane structures also had issues with their weight and folded volume when folded. To address this, the invention of boom–membrane structures was a good way to solve these issues, whose deployment and tension-forming process were driven and supported by appropriate elastic extendable mechanisms, for example, tape spring booms [1,2]. Figure 1 presents a diagram of a deployable tape spring boom. An early application of this kind of boom was in the Canadian satellite “Alouette”, whose booms were made by Klein in the 1960s [3]. The boom extended outwards like a carpenter’s tape measurer, and no hinge was introduced during the whole deployment process, which made this kind of mechanism have a small storage volume after being folded. Under the driving force of the controlling motor connected with the hub, the boom deployed successfully on “Alouette” in space and accomplished the first in-orbit deployment of a tape spring boom. Early deployable booms were mainly made of metal materials, such as steel or CuBe. With the development of the material sciences in the last century, tape spring booms manufactured with composites, especially carbon-/glass-fiber-reinforced polymer (FRP) laminates, were produced and applied [4,5,6,7], as the composite laminate materials gave the booms more design flexibility, higher reliability and better deployment stability [8,9].

Along with the improvement in this new kind of tape spring boom mechanism, boom–membrane structures that could form planar surface space structures appeared afterward. Boom–membrane structures extremely expanded the application area of tape spring booms, which were further used on large-scale solar arrays, solar sails, observation antennas and space telescope star-shaders. Tape spring booms were also used for a 40 m^2^ Synthetic Aperture Radar (SAR) antenna structure developed by DLR and ESA [10,11,12]. During the SAR’s deployment, the beams connected with the boom tips moved outwards under the driving force of the tape spring booms (which were connected by a controlling motor), thus pulling the membrane outwards and stretching and providing tension to the membrane at the end of the deployment. After being fully deployed, the membrane was supported and kept tensioned by the tape spring booms on both sides.

In the 2010s, a prototype of a new boom–membrane solar array was made by the DSS company, called ROSA (Roll-Out Solar Array) because this structure was distinguished from the common types of solar array, like SAR, due to the roll-out deployment type [13,14]. For ROSA, the membrane was coiled on the tip beam when folded and deployed with a roll-out movement of the beam (like opening a scroll), and no controlling motor was needed for the deployment. Because of this, the advantages of the boom–membrane structure were further exploited, and the maximum deployed area was up to 100 m^2^ for each unit. DSS also made the structure modularized, and a higher deployed area can be expected. Since the roll-out deployment type could not provide tension to the membrane, a stretching mechanism needed to be arranged at the root of the structures.

Apart from the advantages mentioned above, compared with traditional deployable truss planar structures, the deployed states of boom–membrane structures had a lower stiffness, buckling load and fundamental frequency. The first formal in-orbit application of a boom–membrane structure was on the Hubble telescope’s solar array, and the structure buckled and failed soon after launch because of strength and vibration issues [15,16,17]. The former research was mainly concentrated on the analysis of the deployment process of tape spring booms and membrane structures, and studies on either the deploying or deployed states were relatively independent [18,19]. Since deployable booms have usually been made from laminate composites in recent years, the changes in the laminate parameters would have a relatively large effect on the properties of both the deploying and deployed states. Therefore, following up on the authors’ previous research [20,21], this paper establishes an analytical model of tape spring booms and boom–membrane structures in their deployed states, and the parametric effect on their deployed properties is deeply investigated. The multi-configuration optimization of the structures’ deploying and deployed states will be further studied in our future work, which will be conducted on the basis of the research in this paper. Section 2 establishes the analytical model of a composite laminate deployable tape spring boom and the corresponding boom–membrane structure, where the boom’s bending stiffness, critical buckling load and the fundamental frequency of the membrane structure are acquired. Section 3 analyzes the parametric effect on the boom’s buckling load and the membrane system’s fundamental frequency to provide more guidance on the boom and the structure design. Further, for the sake of giving some practical verification, the experimental study of the deployed state of the booms and the membrane structures is carried out in Section 4. Section 5 concludes the paper and a discussion is presented in this section.

## 2. Analytical Model Establishment of a Boom–Membrane Structure

In terms of the boom–membrane structures introduced in Section 1, the tape spring booms were commonly made from composite laminate materials, especially for carbon-fiber- or glass-fiber-reinforced polymers. Therefore, the boom material properties should first be analyzed, and the study of the corresponding boom–membrane structures is investigated afterward in this section.

### 2.1. Mathematical Model Establishment

**Figure 2:**
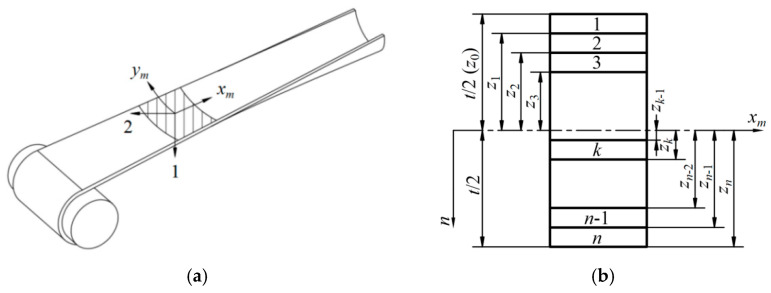
Diagrams of the geometric and laminate parameters of a tape spring boom structure. (**a**) Fiber braided direction (*x_m_* and *y_m_* represent the coordinate system of the boom laminates in which *x_m_* points at the boom’s deployment direction). (**b**) Laminate material parameters (the symbols in this figure were the same as those commonly used in the Classical Laminate Theory, which can be found in Ref. [3]).

For a better understanding of the analysis in this subsection, the diagram of an FRP tape spring boom and the corresponding laminate parameters are given in Figure 2. Generally, fiber-reinforced polymer (FPR) materials consist of one unidirectional (UD) layer in the middle and several fiber layers symmetrically or an-symmetrically distributed on both sides of the UD layer. Note that the model establishment process as follows was carried out under linear hypothesis, and the boom structure was regarded as a thin-walled structure during the whole analysis.

Firstly, according to the theory of materials mechanics, the elastic parameters of the UD layer can be presented through the equations as follows [8]:

(1) $$\left\{\begin{array}{c} E_{1UD}=E_{f}V_{UD}+\left(1-\phi_{UD}\right)E_{m}\left(1-V_{UD}\right)\\ E_{2UD}=\frac{E_{f}\left(1-\phi_{UD}\right)E_{m}}{\left(1-\phi_{UD}\right)E_{m}V_{UD}+E_{f}\left(1-V_{UD}\right)}\\ \nu_{12UD}=\nu_{f}V_{UD}+\nu_{m}\left(1-V_{UD}\right)\\ G_{12UD}=\frac{1}{\frac{V_{UD}}{G_{f}}+\frac{1-V_{UD}}{\left(1-\phi_{UD}\right)G_{m}}} \end{array}\right.$$

where *E*_1UD_ and *E*_2UD_ are the elastic moduli along the fiber direction and the normal direction, respectively (see Figure 2a for more details), *E_f_* and *E_m_* are the elastic moduli of the fiber and the matrix, *V*_UD_ and *ϕ*_UD_ are the fiber volume friction and the porosity coefficient of the UD layer, *ν*_12UD_, *ν_f_* and *ν_m_* are the Poisson’s ratios of the UD layer (overall), the fibers and the matrix, and *G*_12UD_, G*_f_* and *G_m_* are the shear moduli of the UD layer (overall), the fibers and the matrix, respectively.

Secondly, similarly, the parameters of the fiber layer are acquired only through substituting the subscripts in Equation (1), which can be expressed as:

(2) $$\left\{\begin{array}{c} E_{1f}=E_{f}V_{f}+\left(1-\phi_{f}\right)E_{m}\left(1-V_{f}\right)\\ E_{2f}=\frac{E_{f}\left(1-\phi_{f}\right)E_{m}}{\left(1-\phi_{f}\right)E_{m}V_{f}+E_{f}\left(1-V_{f}\right)}\\ \nu_{12f}=\nu_{f}V_{f}+\nu_{m}\left(1-V_{f}\right)\\ G_{12f}=\frac{1}{\frac{V_{f}}{G_{f}}+\frac{1-V_{f}}{\left(1-\phi_{f}\right)G_{m}}} \end{array}\right.$$

**Figure 3:**
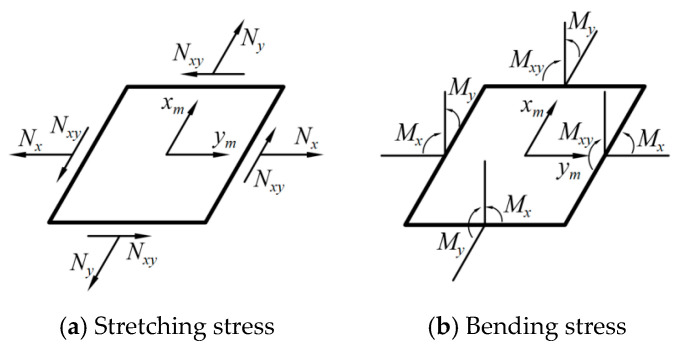
Stress diagrams of laminate composites in deformation (coordinate *x_m_Oy_m_* is the same with that listed in Figure 2a).

According to the Classical Laminate Theory (CLT), the elastic constant of each lamina was obtained through [8] (note that the definitions of the parameters in Equations (1) to (8) were the same as those commonly used in the CLT which would not be re-defined or clarified in this paper. Meanwhile, the corresponding deformation diagrams of FRP composites can be seen in Figure 3 for better understanding):

(3) $$Q=\left[\begin{array}{ccc} Q_{11} & Q_{12} & 0\\ Q_{12} & Q_{22} & 0\\ 0 & 0 & Q_{66} \end{array}\right]$$

in which

(4) $$\left\{\begin{array}{c} Q_{11}=\frac{E_{1}}{1-\nu_{12}\nu_{21}}\\ Q_{22}=\frac{E_{2}}{1-\nu_{12}\nu_{21}}\\ Q_{12}=\frac{\nu_{12}E_{1}}{1-\nu_{12}\nu_{21}}\\ Q_{66}=G_{12}\\ \frac{\nu_{12}}{E_{2}}=\frac{\nu_{21}}{E_{1}} \end{array}\right.$$

and the angle conversion formula was

(5) $$T=\left[\begin{array}{ccc} \cos^{2}\delta & \sin^{2}\delta & 2\sin\delta \cos\delta \\ \sin^{2}\delta & \cos^{2}\delta & -2\sin\delta \cos\delta \\ -\sin\delta \cos\delta & \sin\delta \cos\delta & \cos^{2}\delta -\sin^{2}\delta \end{array}\right]$$

where *δ* is the fiber braided angle in each lamina.

Therefore, the elastic constant of each lamina after the angle converted can be finally expressed as:

(6) $$\bar{Q}=\left[\begin{array}{ccc} {\bar{Q}}_{11} & {\bar{Q}}_{12} & {\bar{Q}}_{16}\\ {\bar{Q}}_{12} & {\bar{Q}}_{22} & {\bar{Q}}_{26}\\ {\bar{Q}}_{16} & {\bar{Q}}_{26} & {\bar{Q}}_{66} \end{array}\right]=T^{-1}Q{\left(T^{-1}\right)}^{T}$$

Moreover, based on the elastic property of each lamina presented in Equation (6), the elastic behaviors of overall laminate composites can be described by the ABD matrix of the CLT, which is commonly shown as:

(7) $$\left[\begin{array}{c} N\\ M \end{array}\right]=\left[\begin{array}{cc} A & B\\ B & D \end{array}\right]\left[\begin{array}{c} \varepsilon \\ \kappa \end{array}\right]$$

which might be expressed more specifically as:

(8) $$\left[\begin{array}{c} N_{x}\\ N_{y}\\ N_{xy}\\ M_{x}\\ M_{y}\\ M_{xy} \end{array}\right]=\left[\begin{array}{cccccc} A_{11} & A_{12} & A_{16} & B_{11} & B_{12} & B_{16}\\ A_{12} & A_{22} & A_{26} & B_{12} & B_{22} & B_{26}\\ A_{16} & A_{26} & A_{66} & B_{16} & B_{26} & B_{66}\\ B_{11} & B_{12} & B_{16} & D_{11} & D_{12} & D_{11}\\ B_{12} & B_{22} & B_{26} & D_{12} & D_{22} & D_{26}\\ B_{16} & B_{26} & B_{66} & D_{16} & D_{26} & D_{66} \end{array}\right]\left[\begin{array}{c} \varepsilon_{x}\\ \varepsilon_{y}\\ \gamma_{xy}\\ \kappa_{x}\\ \kappa_{y}\\ \kappa_{xy} \end{array}\right]$$

Furthermore, the elements in the ABD matrix in Equation (8) can be acquired by:

(9) $$\left\{\begin{array}{c} A_{ij}=\sum_{k=1}^{n}{\left({\bar{Q}}_{ij}\right)}_{k}\left(m_{k}-m_{k-1}\right)\\ B_{ij}=\frac{1}{2}\sum_{k=1}^{n}{\left({\bar{Q}}_{ij}\right)}_{k}\left(m_{k}^{2}-m_{k-1}^{2}\right)\\ D_{ij}=\frac{1}{3}\sum_{k=1}^{n}{\left({\bar{Q}}_{ij}\right)}_{k}\left(m_{k}^{3}-m_{k-1}^{3}\right) \end{array}\right.$$

where *n* is the number of laminate layers on either side of the UD layer (apart from the UD layer) and *m_k_* is the distance between the neutral surface and the outer side of layer *k* (see Figure 2b) which can be further described by:

(10) $$m_{k}=kt_{f}+\frac{t_{UD}}{2}$$

in which *t*_UD_ and *t_f_* are the thicknesses of the UD layer and the fiber layer (commonly, the thicknesses of the fiber layers using one boom structure were the same).

### 2.2. Boom Bending Property Calculation

**Figure 4:**
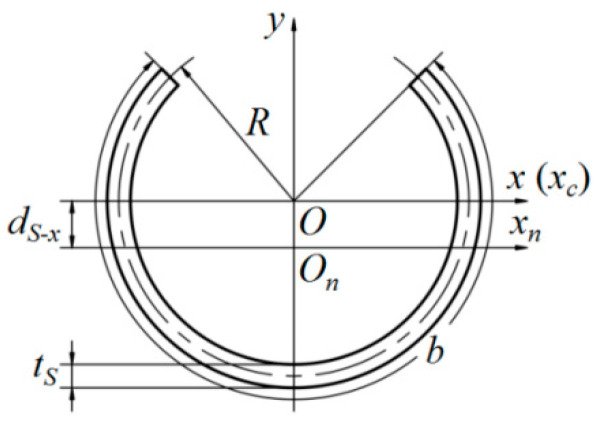
Diagram of boom transversal cross-section configuration.

For the sake of illustration, the diagram of a tape spring boom (transversal) cross-section configuration is shown in Figure 4. According to the deployment mode of the boom–membrane structures, the bending torque acted on the boom (after deployment) was mainly around the *x* or *y*-axis shown in Figure 4. Hence, the expressions of the bending stiffness around the *x* and *y*-axes would be, respectively, studied as follows. Note that *O* is the center of the boom geometric circle while *O_n_*-*x_n_* is the neutral axis of the boom cross-section configuration in Figure 4.

Based on the geometric configuration shown in Figure 4, the boom’s moment of inertia around the *x_c_*-axis can be seen to be:

(11) $$I_{x_{c}}=2\int_{R-\frac{t_{S}}{2}}^{R+\frac{t_{S}}{2}}R_{i}dR_{i}\int_{0}^{\frac{b}{2R}}R_{i}^{2}\sin^{2}\theta d\theta$$

where *R_i_* is the variable of integration along radius *R*, *t_S_* is the total thickness of the boom structure, and *θ* is the angular variable of integration under a polar coordinate.

On the basis of the parallel-axis formula, the moment of inertia around the *x_n_*-axis is:

(12) $$I_{x_{n}}=I_{x_{c}}-d_{S-x}^{2}H_{S}$$

in which *H_s_* is the total area of the boom cross-section configuration and *d_S_*_-*x*_ showed the distance between *x_c_* and *x_n_*-axes, which can be acquired, respectively, through the equations as follows:

(13) $$H_{S}=2\int_{R-\frac{t_{S}}{2}}^{R+\frac{t_{S}}{2}}R_{i}dR_{i}\int_{0}^{\frac{b}{2R}}d\phi$$

(14) $$d_{S-x}=\frac{2\int_{R-\frac{t_{S}}{2}}^{R+\frac{t_{S}}{2}}R_{i}dR_{i}\int_{0}^{\frac{b}{2R}}R_{i}\sin\phi d\phi }{H_{S}}$$

Substituting Equations (13) and (14) into Equation (12) and combining Equations (8) and (12), the boom’s bending stiffness around the *x*-axis can be finally acquired as:

(15) $$S_{x}=\frac{A_{11}I_{x_{n}}}{t_{S}}$$

Similarly, from the diagram in Figure 4, the moment of inertia and the bending stiffness around the *y*-axis (the *y*-axis is just the boom’s neutral axis in this bending mode) were:

(16) $$I_{y}=2\int_{R-\frac{t_{S}}{2}}^{R+\frac{t_{S}}{2}}R_{i}dR_{i}\int_{0}^{\frac{b}{2R}}R_{i}^{2}\cos^{2}\phi d\phi$$

(17) $$S_{y}=\frac{A_{11}I_{y}}{t_{S}}$$

### 2.3. Boom Buckling Load Calculation

For a boom–membrane structure working on-orbit, the boom’s buckling failure usually results from a normal tip load caused by the tension requirement of the membrane. According to Equations (15) and (16) and the Euler buckling formula, the critical buckling load of the tape spring boom is:

(18) $$P_{cr}=\frac{\pi^{2}}{4l^{2}}\cdot \min\left\{S_{x},S_{y}\right\}$$

where *l* is the total length of the boom. Note that the boundary conditions for the boom buckling calculation is as follows: the boom root is fully fixed with total a free tip (i.e., one end fixed and another end free for each boom), and the load acted (pressed) along the boom length.

### 2.4. Fundamental Frequency Calculation of Boom–Membrane Structures

On the basis of Refs. [18,19], the fundamental frequency of the boom–membrane structure can be obtained through the formula as shown:

(19) $$f=\frac{1}{2\pi }\sqrt{\frac{n_{b}S}{l^{3}\left(0.2235\cdot \left(p_{m}q_{m}\rho_{ma}+n_{b}lH_{s}\rho_{b}\right)+l_{r}\rho_{rl}\right)}}$$

where *n_b_* is the number of the tape spring booms included in a boom–membrane structure, *S* is each boom’s bending stiffness (the stiffness of each boom is set as the same), *p_m_* and *q_m_* are the length and the width of the membrane, respectively, *ρ_ma_* and *ρ_b_* are the densities of the membrane and the tape spring boom (on average), *H_s_* is the area of one boom’s cross-section (the same with that used in Equation (12)), *l_r_* is the length of the tip beam, and *ρ_ri_* is the linear density of the tip beam.

Note that Equation (19) only aims to acquire the frequency of the modal when the structure is swinging around the root beam (which is the weakest stiffness direction), and the slits of the tape spring booms are commonly in the normal direction of the membrane because of structural limits (hence, *S* = *S_x_* in Equation (19)). A limitation of the analysis above is that the deformation of the booms should be relatively small, and this condition is in accordance with the common working situations of a boom (used in a membrane structure) working in space.

## 3. Parametric Study of Tape Spring Booms and Boom–Membrane Structures

To provide more guidance on the boom–membrane structure design, a parametric study is carried out in this section. Table 1 presents the geometric and material parameters of an FPR composite boom which was used for mimicking those used for InflateSail launched in the year 2015 [22,23], while Table 2 lists the parameters of the corresponding boom–membrane structure made of the booms with the parameters shown in Table 1.

Generally, for a boom–membrane structure working in space, the tape spring boom’s critical buckling load and the membrane structure’s fundamental frequency had great influences on the system’s performance. Meanwhile, the effect of a tape spring boom’s parameters is sensitive and complicated. Hence, the parametric study of a boom's geometric and material parameters is presented as follows. Note that the parameters of the two booms in a boom–membrane structure can be changed simultaneously in the analysis in this section.

### 3.1. Study of Boom Geometric Parameters

**Figure 5:**
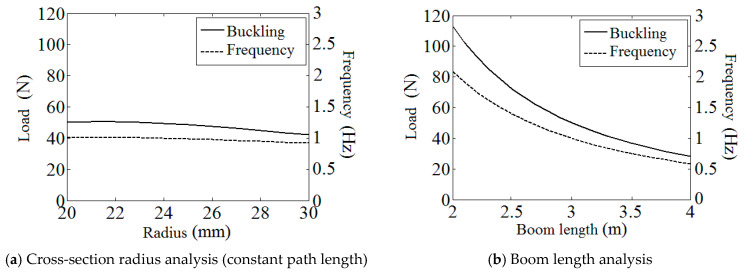
Boom geometric parametric study.

Figure 5 presents the variations in the boom critical buckling load and the membrane structure fundamental frequency under the changing of the boom’s cross-section radius *R* (with a constant path length *b*) and the total boom length *l*, respectively. Note that the left vertical axis in each sub-figure is for buckling load (N) while the right axis presents system frequency (Hz), and the parameters that are not marked in the figures are the same as those listed in Table 1 and Table 2.

On the basis of the plots in Figure 5a, it can be observed that both the buckling load and the fundamental frequency decreased with the increase in the boom radius. Meanwhile, generally, these two indicators are less sensitive to the radius change (the boom’s bending stiffness changes with the changing of the boom’s cross-section radius and the buckling load and the fundamental frequency vary with the change in the bending stiffness). This is because higher *R* (with a constant path length *b*) made the boom cross-section flatter, which made the boom more vulnerable to bending torques, and at the same time, the increase in *R* made the boom’s cross-section more scattered, which could partly compensate for the effect mentioned above. From Figure 5b, the load and frequency plots also descend with a growing boom length, and this trend can be relatively more sensitive when the boom is relatively short. This performance is mainly caused by the increase in total mass and the moving outwards of the structure barycenter.

### 3.2. Study of Boom Laminate Parameters

**Figure 6:**
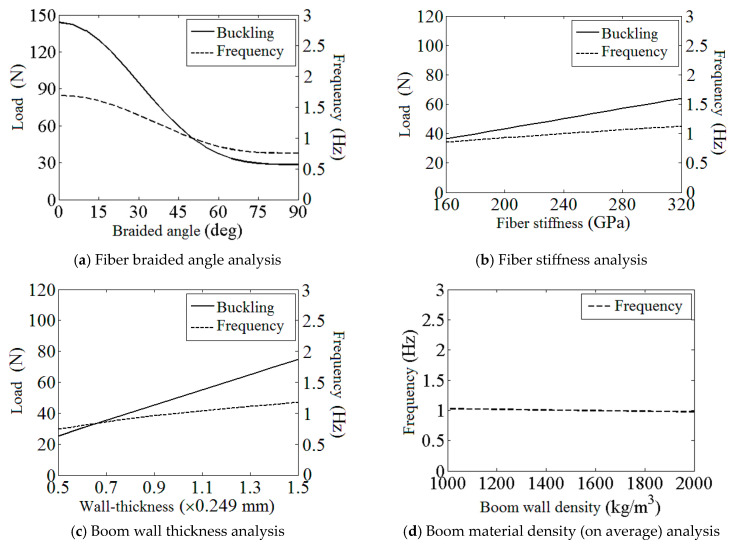
Boom laminate parametric study.

In Figure 6, the influence of the boom laminate parameters, such as the fiber braided angle, the fiber stiffness, the boom wall thickness (overall), and the boom material density (on average), on the boom’s buckling load and the membrane structure’s fundamental frequency is shown. Note that in Figure 6a, only the braided angle of the fiber layers is changed (every lamina has the same absolute angle value during the analysis) and the angle of the UD layer is always kept constant at 0°. Moreover, as the density has no connection with the boom’s buckling load, only the frequency plot is shown in Figure 6d.

According to the results listed in Figure 6a,b, the buckling load and the fundamental frequency increase with the decreasing of the braided angle or the increase in the fiber stiffness. This is due to the parametric change above enhanced the structural strength and the stiffness along the boom length (direction 1 in Figure 2a). For the plots in Figure 6c, the volume fractions of each lamina were kept constant under the change in the boom’s total wall thickness. From Figure 6c, the rise in the wall thickness enhances the tape spring boom’s stiffness (this effect also improves the boom’s critical buckling load), yet adds the boom’s total mass at the same time as well. Therefore, the frequency increases slowly with a growing wall thickness in the plot under these combined actions. Further, it can be seen in Figure 6d that the variation in the boom material density has little effect on the membrane structure’s frequency. Therefore, from the result in Figure 6d, reducing the boom density is not an effective way to improve the dynamic stiffness of the boom–membrane structure.

## 4. Experimental Study

For the sake of providing some practical verification to the theoretical analysis in the above sections, the experiment of the tape spring boom bending stiffness test and the boom–membrane structure frequency test were carried out. Since the boom buckling test is a kind of damage experiment and the critical load directly depends on the boom’s bending stiffness according to the Euler formula (Equation (18)), the boom buckling experiment is not necessary for verifying the research in this paper.

### 4.1. Boom Bending Stiffness Experiment

**Figure 7:**
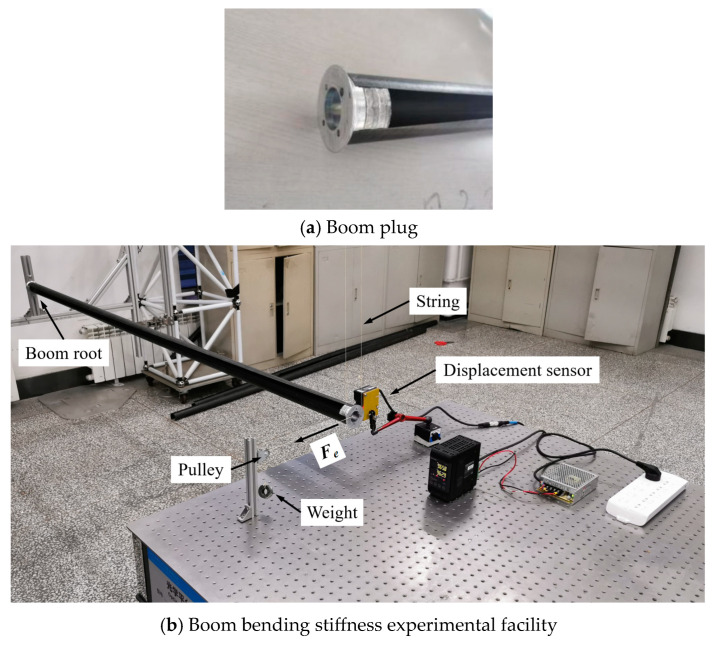
Boom bending stiffness experiment.

The parameters of the tape spring boom sample used for the experiment were *R* = 25 mm, *b* = 137.5 mm, *l* = 2000 mm and the boom’s laminate layout was set as [±50°F/0°]_S_ (carbon-fiber-reinforced polymers) in order to better manufacture (the other parameters were the same with those listed in Table 1). For keeping the configurations of the tip and the root of the boom sample during the bending test process, customized plugs were introduced at the tip and the root of each sample (see Figure 7a).

**Figure 8:**
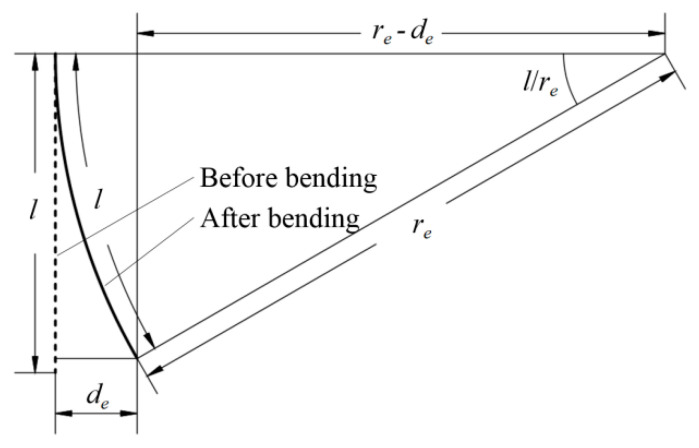
Diagram of boom bending stiffness conversion.

Figure 7b shows the facilities used for the single-boom bending stiffness experiment. In the figure, the boom root was fully fixed (all DOFs restrained) while the boom tip was hanging up on the ceiling with a string for compensating the boom and the plug gravity. The tip was loaded by weights through a fixed pulley, and the tip displacement was acquired and recorded by a laser displacement sensor which was fixed on the test bed (fixed on the ground). The value of the counterweight was selected on the premise of small deformation of the boom. Further, the boom tip displacement and the counterweight was converted into bending stiffness (*S_e_*) through the following equations (see Figure 8 for the corresponding conversion diagram):

(20) $$\left\{\begin{array}{c} {\left(r_{e}-d_{e}\right)}^{2}+{\left(r_{e}\cdot \sin\left(\frac{l}{r_{e}}\right)\right)}^{2}=r_{e}^{2}\\ S_{e}=M_{e}\cdot r_{e} \end{array}\right.$$

where *M_e_* is the bending torque acted on the boom tip caused by the weights (see Figure 8 for other symbol definitions in Equation (20)). Before the beginning of the experiment, the laser displacement sensor was calibrated and reset as zero for the measurements, and the results obtained from the laser sensor after the experiment can be combined with the counterweight values based on the conversion in Figure 8 and Equation (20).

The boom bending stiffness experiment results are listed in Table 3, and the stiffness around *x* and *y*-axes were listed and compared with the corresponding theoretical results, respectively. According to the comparison, it can be observed that the testing and analytical results matched with each other very well, and all the experimental relative errors were below 2%. Meanwhile, a higher counterweight led to lower testing errors as appropriate weight made the boom bending more ideal. To sum up, from the testing experiment it can be known that the analytical method in this paper is available for solving the bending stiffness and the buckling load of an FRP composite laminate tape spring boom.

### 4.2. Fundamental Frequency Experiment of Boom–Membrane Structure

**Figure 9:**
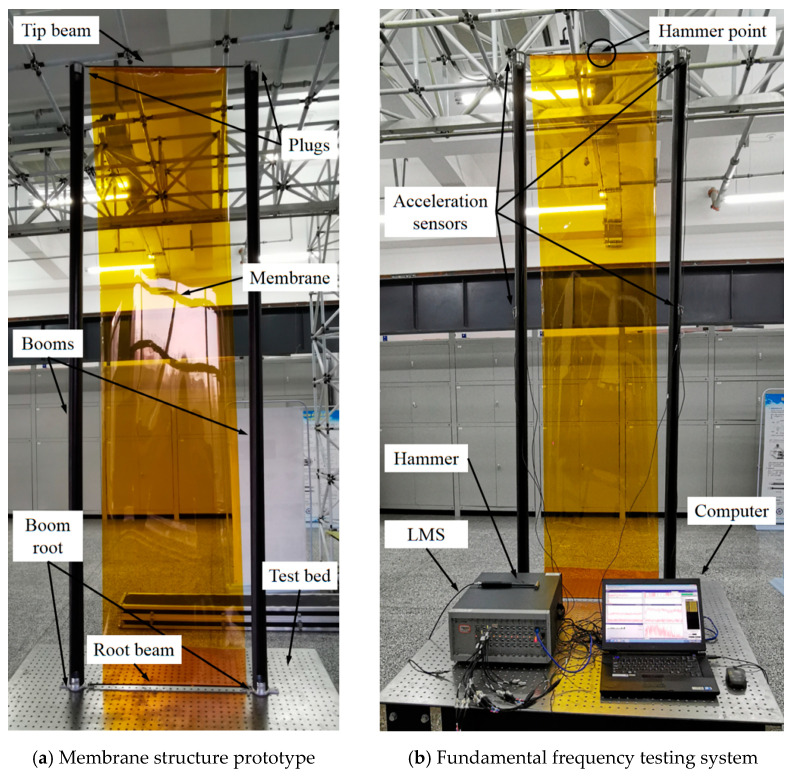
Membrane structure prototype and fundamental frequency experimental facility.

In the fundamental frequency experiment, the geometric parameters of the boom–membrane structure prototype were changed to the values presented in Table 4 for the ease of manufacturing (the other parameters were in accordance with those values in Table 2). Based on the corresponding parameters listed in Table 4, the boom–membrane structure prototype is made and shown in Figure 9a. Figure 9b presents the fundamental experimental facilities. In the figures, the roots of the two tape spring booms were fixed on the test bed (all DOFs restrained). The boom tips were connected with a tip beam (plugs introduced on each boom’s tip and root as shown in Figure 7a and the weight of each plug was measured as 0.5 kg), and the membrane (made from polyimide) was tensioned between the tip beam and the root beam close to the test bed. The membrane was tensioned between the root and tip beams, while the acceleration sensors were assigned and attached along the tape spring booms sending the testing data to the LMS vibration testing system (developed by Siemens). Through the LMS Scadas III Test Lab resolving software and interface, the vibration frequency of the boom–membrane structural prototype can be displayed on the computer. The hammer point was selected at the middle of the tip beam based on the previous testing experience. To improve the reliability of the experimental results, four tests were carried out during the frequency experiment. Before the fundamental frequency experiment, the LMS vibration testing system was calibrated by testing a steel plain with known vibration properties. During the test, the boom–membrane structural prototype stood on the test bed (which was fully fixed on the ground) like a vertical cantilever with its two boom roots and one root beam wholly restrained on the test bed (see Figure 9), which was used for mimicking the zero-gravity condition when working on-orbit. The damping factor was ignored for the experiment since the oscillating velocity was relatively low; hence, the air-damping and the structural damping were negligible.

The experimental results of the membrane structure’s fundamental frequency are shown in Table 5, and the corresponding theoretical results were also listed for comparison. On the basis of the results in Table 5, it can be seen that the experimental results generally matched with the theory, and the errors of the four tests were all below 10%. Furthermore, from Table 5, experimental results were slightly higher than the theory. This was because the structure stood on the test bed; hence, the gravity component would reduce the system’s vibration frequency. Nevertheless, this experiment could verify that the theory in this paper is available for predicting the fundamental frequency of this kind of boom–membrane structure. By comparing with the investigation in Refs. [24,25], the method in this paper better balanced the model complexity and the result accuracy for acquiring the properties of the FRP composite booms and the corresponding boom–membrane structures.

## 5. Conclusions and Discussion

Extendable deployable tape spring boom mechanisms were commonly used for extra large-scale space structures which were usually combined with flexible membranes forming planar boom–membrane structures, which can meet the needs of large planar space structures. The early tape spring booms were usually made from metal materials, while FRP laminate composites have often been used to improve the stability and design flexibility of the booms recently. However, the research of the previous work is mainly concentrated on the boom deploying process, and the properties of the deployed and deploying states were usually studied independently.

The work of this paper aimed to analyze the deployed state of the FRP composite laminate tape spring booms and the corresponding boom–membrane structures. For this purpose, the analytical models of the laminate tape spring boom and the boom–membrane structure were established to solve the boom’s bending stiffness, buckling loads and the membrane structure’s fundamental frequency. For the sake of understanding the influence of the key design parameters, a parametric study was carried out to afford more guidance for a better design. Based on the geometric parameter analysis, it can be found that the buckling and frequency properties are relatively insensitive to the boom’s cross-section radius change (with a constant path length) while they are sensitive to the boom’s total length. On the basis of the laminate parametric study, both of the indicators grow with the enhancement of the boom bending stiffness and the boom wall thickness.The boom material density has a slight effect on the system’s fundamental frequency. To provide some practical verification, the experiment on a tape spring boom and the boom–membrane structure was carried out. Comparing the stiffness and frequency results from the experiment and the theory, it can be observed that the analytical method in this paper is available for predicting the deployed properties of the booms and the structures.

Furthermore, in the design of deployable structure mechanisms, the properties of the structure in both deploying and deployed states should be considered comprehensively. The work in this paper follows up on the research of the authors’ previous study which aimed at the boom–membrane structure deploying process. The multi-configuration optimization of the structure will be further researched and published in the near further, which will be on the basis of the work in this paper.

## Figures and Tables

**Table 1:** Geometric and material parameters of tape spring booms.

*R* (mm)	*b* (mm)	*E_m_* (GPa)	*G_m_* (GPa)	*ν_m_*	*E_f_* (GPa)
20	110	4	2.7	0.35	240
*G_f_* (GPa)	*ν_f_*	*t*_UD_ (mm)	*V*_UD_ (%)	*ϕ*_UD_ (%)	*t_f_* (mm)
95	0.22	0.057	31	15	0.096
*V_f_* (%)	*ϕ_f_* (%)	*μ*	*E_h_* (GPa)	*E_r_* (GPa)	Lay out
53	15	0.1	205	205	[±50°F/0°]_s_ *

* Mark “F” meant fabric laminate layout.

**Table 2:** Parameters of boom–membrane structure.

Membrane Size *p_m_* × *q_m_*	Membrane Areal Density *ρ_ma_*	Boom Length *l*	Boom Distance *q_m_*	Boom Density (On Average) *ρ_b_*	Tip Beam Length *l_r_*	Tip Beam Linear Density *ρ_rl_*
2950 × 900 mm^2^	0.3 kg/m^2^	3000 mm	1000 mm	1500 kg/m^3^	1100 mm	0.1 kg/m

**Table 3:** Experiment results of boom bending stiffness.

Weight (g)			5	10	15	20
Corresponding torque (N∙m)			0.98	1.96	2.94	3.92
Around *x*-axis	Displacement (mm)		11.46	22.92	34.39	45.88
	Bending stiffness	Test (N∙m^4^)	349.0	349.0	348.9	348.7
		Theory (N∙m^4^)	343.4			
		Error (%)	−1.63	−1.63	−1.60	−1.54
Around *y*-axis	Displacement (mm)		8.12	16.29	24.56	32.98
	Bending stiffness	Test (N∙m^4^)	492.6	491.1	488.6	485.1
		Theory (N∙m^4^)	484.3			
		Error (%)	−1.71	−1.40	−0.98	−0.17

**Table 4:** Parameters of boom–membrane structure used for experimental study.

Membrane size *p_m_* × *q_m_*	Membrane areal density *ρ_ma_*	Boom length *l*	Boom distance *q_m_*
1980 × 450 mm^2^	0.15 kg/m^2^	2000 mm	570 mm
Boom density (on average) *ρ_b_*	Tip beam length *l_r_*	Tip beam linear density *ρ_rl_*	Membrane tension *T_m_*
1500 kg/m^3^	620 mm	0.05 kg/m	10 N

**Table 5:** Experimental results of boom–membrane structure fundamental frequency.

	Test 1	Test 2	Test 3	Test 4
Experiment (Hz)	3.08	3.26	3.10	3.15
Theory (Hz)	3.32			
Error (%)	+7.8	+1.8	+7.1	+5.4

## Data Availability

The data presented in this study are available on request from the corresponding author. The data are not publicly available due to relevant policy restrictions.
